# A hundred days into the coronavirus disease (COVID-19) pandemic

**DOI:** 10.2807/1560-7917.ES.2020.25.14.2000550

**Published:** 2020-04-09

**Authors:** Ines Steffens

**Affiliations:** 1 Editor-in-chief Eurosurveillance, European Centre for Disease Prevention and Control (ECDC), Stockholm, Sweden

**Keywords:** Pandemic, corona virus disease, COVID-19, world, SARS-CoV-2, severe acute respiratory syndrome coronavirus, social distancing

On 8 April 2020, 100 days have passed since ProMed posted a ‘request for information’ about the emergence of a cluster of viral pneumonia of unknown origin in Wuhan [1]. Back then, a number of similarities triggered memories of the beginning of the severe respiratory syndrome (SARS) epidemic caused by the SARS coronavirus (SARS-CoV) in 2003 [2]. Today, the ProMed post of 30 December 2019 appears far back in time; the coronavirus disease (COVID-19) caused by SARS-CoV-2 has far exceeded the 2003 SARS epidemic in terms of magnitude and impact. Since the beginning of January 2020, weeks and days have been dominated by changes that had a major bearing on people’s professional and personal lives; changes in the epidemic, changes in knowledge about the virus and the disease, and changes in public health measures at a scale that only months ago were inconceivable.

## Epidemic evolution

In January and February, the worldwide focus of attention was China where cases and fatalities increased day by day. Just 30 days after ProMed’s post, on 29 January, 6,065 confirmed COVID-19 cases had been notified worldwide, mainly from China, where all 1,239 severe cases and 132 COVID-19 deaths were reported. At that point, only 68 cases had been identified in 15 countries outside mainland China, all of which had a link to China or to cases from China [3] (Figure). By 28 February, 30 days later, 83,652 confirmed COVID-19 cases had been reported from 51 countries on all continents. Countries outside mainland China still accounted for only 4.5% (n = 3,664) of the notified cases with some, including Italy, Germany, and France in Europe, already experiencing limited autochthonous transmission. From late February, new infections in China declined rapidly after the implementation of sweeping social distancing measures, while attention turned to Europe, the new epicentre of the COVID-19 pandemic. Italy was initially the country hit the hardest by far, Spain, the Netherlands and others followed; France and Germany had experienced the first importation of cases already in January [4,5]. On 10 March, Italy passed the mark of 10,000 confirmed cases and on 19 March, the total number of fatalities in Italy exceeded 3,000, topping the total number of reported fatalities in China. Cases with a link to Italy and neighbouring regions of Austria were detected in a number of European countries in February and early March, often linked to tourists returning from skiing holidays [6]. Outside Europe, Iran faced a rapid surge of COVID-19 followed by exportation of cases mostly to countries in the Middle East but also overseas [7]. More recently, the United States in North America, and in Europe the United Kingdom emerged as new epidemic hotspots with 124,655 cases plus 2,191 fatalities and 17,089 cases plus 1,019 fatalities, respectively, reported by 29 March, day 90 after the ProMed post [3]. Recently, increasing case numbers have also been seen in Africa and in Asian countries outside China that had not been affected early in the pandemic. On 8 April, day 100 after ProMed’s ‘request for information’, 1,391,890 cases including 81,478 fatalities have been reported from more than 200 countries/territories worldwide and only few countries appear to have passed the peak.

**Figure:**
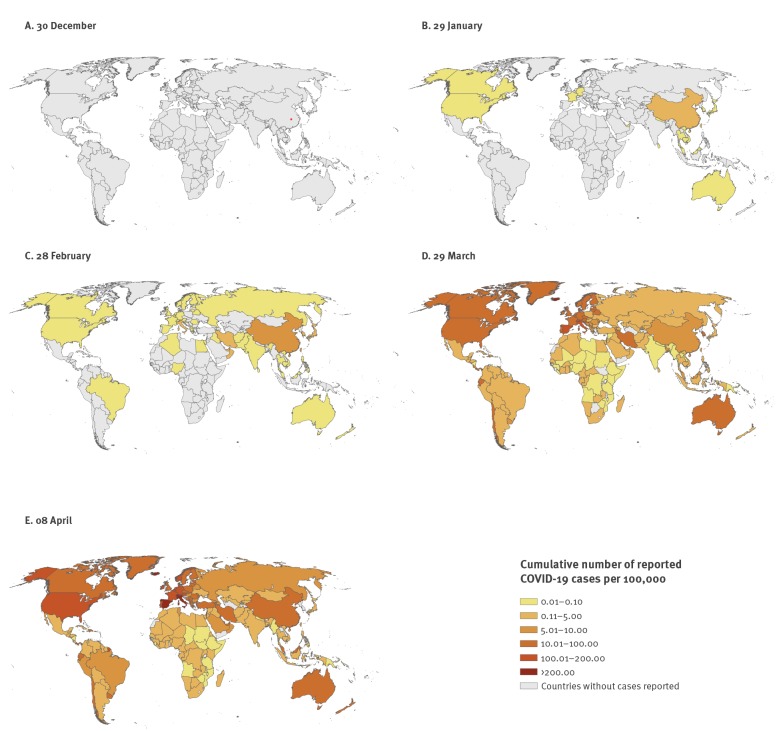
Reported COVID-19 cases per 100,000 population by country over 100 days, 30 December 2019–8 April 2020 (n = 1,391,890)

An animation of the cumulative number of reported of COVID-19 cases per 100,000 population is provided in the supplementary material (Video S1).

On 31 December 2019, 27 cases were officially reported by the Hubei Provincial Health and Health Commission [1]. The red asterisk in panel A indicates the location of Wuhan, Hubei Province, China.

Source: European Centre for Disease Prevention and Control. The boundaries and names indicated on this map do not imply official endorsement or acceptance by the European Union.

International organisations such as the European Centre for Disease Prevention and Control have used novel technologies and open access resources as well as official reporting data to trace the evolution of the COVID-19 pandemic. For the first time, this has enabled updating public health practitioners, policymakers, infectious disease specialists and the general public on the numbers of pandemic cases in close to real time and with high resolution.

## Knowledge gains and remaining questions

The virus causing COVID-19 was identified and announced on 7 January and by 10 January, sequences of five genomes had been shared, providing scientists around the world with a basis for development of diagnostic tests [8]. There was fast growing knowledge about several important parameters for understanding the spread of disease. A number of studies in various settings determined the basic reproductive number R_0_ to be within the range of 2–3 [9,10]; the incubation period was determined as ranging from 1 to 14 days with an average of 5–6 days; transmission through droplets was established as the main mode of transmission; the clinical picture that emerged included a wide range from mild symptoms with fever, sore throat and cough to severe pneumonias with characteristic features on computed tomography scans; in Hubei, China, up to 13.8% of laboratory-confirmed patients had severe disease, 6.1% were critically ill and the case fatality rate among all COVID-19 cases was 2.3% [11].

Further questions on topics such as the possibility of asymptomatic transmission, the proportion of asymptomatic infections, stability of the virus in the environment and main risk groups [12] were partly answered as the pandemic progressed. Evidence of asymptomatic transmission was documented in several settings and a study based on an outbreak on a cruise ship determined the proportion of asymptomatic infections as 17.9% (95% credible interval: 15.5–20.2) [13]. Laboratory experiments provided data on the survival of the virus on different surfaces [14]. Aside from people older than 70 years, severe and fatal cases were seen particularly in men and people with hypertension, diabetes, underlying pulmonary diseases and in smokers. Children were shown to be infected with SARS-CoV-2 [15], however, the role of children in disease transmission still needs to be fully elucidated.

The focus of ongoing research includes determining the duration of virus shedding and infectiousness. Development of serological tests with good sensitivity and specificity for wide-scale use is necessary to assess population immunity. Serological studies have been started in several European countries to define population immunity and studies on the duration of immunity will need to be conducted in the future. Clinical trials are being conducted worldwide to investigate treatment options with a variety of antiviral and other drugs. The World Health Organization (WHO) and European Union-funded projects are coordinating multi-centre adaptive trials to assess the effectiveness of remdesivir, hydroxychloroquine and a combination of lopinavir and ritonavir with or without interferon-beta-1a [16,17]. A number of vaccine trials are ongoing worldwide with earliest results expected within the coming 18 months.

A number of questions and challenges that arose in the COVID-19 pandemic appear similar to those experienced during the 2009 influenza A(H1N1)pdm09 pandemic. However, information sharing by scientists was faster in 2020, also facilitated by international organisations, widespread social media use and policies of a number of scientific journals, including *Eurosurveillance*, that enabled fast-tracking of peer-reviewed articles, publishing of articles already deposited in pre-print servers, and making articles open access. In particular, the global initiative on sharing all influenza data (GISAID) has allowed organisations like nextstrain.org to collectively perform analysis in almost real time.

## Measures and challenges

The WHO declared COVID-19 a Public Health Emergency of International Concern (PHEIC) on 30 January and a pandemic on 11 March [18,19]. In response to the COVID-19 pandemic, countries worldwide have taken to a wide range of measures, initially to contain and later to mitigate its impact. The current goal is primarily to limit the spread of the virus through physical distancing i.e. lowering contact rates in order to gain time for preparing critical services such as healthcare for a high demand [20]. Reducing exposure also aims to protect the most vulnerable population groups and healthcare workers in the absence of a vaccine and specific treatment. In Europe, measures have comprised closure of borders, closure of educational institutions such as schools, nurseries and universities, closure of museums and theatres, closure of shops and restaurants, restriction of movement and suspension of public gatherings even with small groups of people, and lockdown of whole countries. Obviously, these measures have a far-reaching impact on individuals and society as a whole and they were taken at different times and on a different scale depending on the countries’ epidemiological, geographical, political and societal situation. The COVID-19 pandemic has posed a number of public health challenges and some vulnerabilities in health sector preparedness have become evident. In times of public health emergencies, setting research priorities and conducting research risks duplication of efforts, and strong coordinating mechanisms and oversight are necessary to avoid this. Further, timely communication and conveying uncertainties are a recurrent challenge for public health and policymakers. Informed risk communication, streamlining of messages and collaboration with the media play an important role in mitigating the impact of any public health crisis. A new challenge of the COVID-19 pandemic is how to identify the right moment and determine the correct means for escalation and de-escalation of measures taken at a scale that was beyond imagination for many only months ago. While there will be common principles such as timeliness, proportionality and acceptability of measures, there will most probably not be one gold standard solution for all and the approaches will need to be flexible and responsive. Only the future can show which strategies and measures were most successful in curbing the pandemic.

It has taken China around 100 days to start getting back to ‘normal’ and early signals from some countries seem to indicate that the increase of new infections may have slowed down. However, the majority of countries worldwide are just beginning to see a surge in cases and excess all-cause mortality is high in several of the European countries most affected by COVID-19 [21]. While it is yet impossible to predict where we will be 100 days from now, COVID-19 will certainly still be on our agenda and there is no room for complacency.

## Supplementary Data

176000 Supplement

## References

[1] Program for Monitoring Emerging Diseases (Pro-MED). Undiagnosed pneumonia - China (HU): Request For Information. Archive Number 20191230.6864153. 30 Dec 2019. Available from: https://promedmail.org/promed-post/?id=6864153

[2] Mackay IM. Viral pneumonia cluster in Wuhan, central China: 44 cases and counting. Brisbane: Virology Down Under; 3 Jan 2020. Available from: https://virologydownunder.com/viral-pneumonia-cluster-in-wuhan-central-china-44-cases-and-counting/

[3] World Health Organization (WHO). Situation reports. Geneva: WHO; 29 Mar 2020. Available from: https://www.who.int/docs/default-source/coronaviruse/situation-reports/20200329-sitrep-69-covid-19.pdf?sfvrsn=8d6620fa_8

[4] Bernard Stoecklin S Rolland P Silue Y Mailles A Campese C Simondon A First cases of coronavirus disease 2019 (COVID-19) in France: surveillance, investigations and control measures, January 2020. Euro Surveill. 2020;25(6):2000094 10.2807/1560-7917.ES.2020.25.6.2000094 PMC7029452 32070465

[5] Bayerisches Staatsministerium fur Gesundheit und Pflege (STMGP). Bestätigter Coronavirus-Fall in Bayern – Infektionsschutzmaßnahmen laufen. [Confirmed coronavirus case in Bavaria – Infection protection measures ongoing]. Munich: STMGP; 27 January 2020. Available from: https://www.stmgp.bayern.de/presse/bestaetigter-coronavirus-fall-in-bayern-infektionsschutzmassnahmen-laufen/

[6] European Centre for Disease Prevention and Control (ECDC). Outbreak of novel coronavirus disease 2019 (COVID-19): increased transmission globally – fifth update. Stockholm: ECDC; 2 March 2020. Available from: https://www.ecdc.europa.eu/en/publications-data/rapid-risk-assessment-outbreak-novel-coronavirus-disease-2019-covid-19-increased

[7] Center for Infectious Disease Research and Policy (CIDRAP). COVID-19 cases surge in South Korea, Italy, and Iran: Minneapolis: CIDRAP; 1 March 2020. Available from: https://www.cidrap.umn.edu/news-perspective/2020/03/covid-19-cases-surge-south-korea-italy-and-iran

[8] Corman VM Landt O Kaiser M Molenkamp R Meijer A Chu DKW Detection of 2019 novel coronavirus (2019-nCoV) by real-time RT-PCR. Euro Surveill. 2020;25(3):2000045. 10.2807/1560-7917.ES.2020.25.3.2000045 31992387 PMC6988269

[9] Liu Y Gayle AA Wilder-Smith A Rocklöv J The reproductive number of COVID-19 is higher compared to SARS coronavirus. J Travel Med. 2020;27(2):taaa021. 10.1093/jtm/taaa021 32052846 PMC7074654

[10] Zhang S Diao M Yu W Pei L Lin Z Chen D Estimation of the reproductive number of novel coronavirus (COVID-19) and the probable outbreak size on the Diamond Princess cruise ship: A data-driven analysis. Int J Infect Dis. 2020;93:201-4. 10.1016/j.ijid.2020.02.033 32097725 PMC7110591

[11] World Health Organization (WHO). Report of the WHO-China joint mission on coronavirus disease 2019 (COVID-19). Geneva: WHO; 2020. Available from: https://www.who.int/docs/default-source/coronaviruse/who-china-joint-mission-on-covid-19-final-report.pdf

[12] Cowling BJ, Leung GM. Epidemiological research priorities for public health control of the ongoing global novel coronavirus (2019-nCoV) outbreak. Euro Surveill. 2020;25(6):2000110. 10.2807/1560-7917.ES.2020.25.6.2000110 PMID:32046814 PMC7029449 32046814

[13] Mizumoto K Kagaya K Zarebski A Chowell G Estimating the asymptomatic proportion of coronavirus disease 2019 (COVID-19) cases on board the Diamond Princess cruise ship, Yokohama, Japan, 2020. Euro Surveill. 2020;25(10):2000180. 10.2807/1560-7917.ES.2020.25.10.2000180 32183930 PMC7078829

[14] van Doremalen N Bushmaker T Morris DH Holbrook MG Gamble A Williamson BN Aerosol and Surface Stability of SARS-CoV-2 as Compared with SARS-CoV-1. N Engl J Med. 2020. [Epub ahead of print] 10.1056/NEJMc2004973 32182409 PMC7121658

[15] Lu X Zhang L Du H Zhang J Li YY Qu J SARS-CoV-2 infection in children. N Engl J Med. 2020; [Epub ahead of print]. 10.1056/NEJMc2005073 32187458 PMC7121177

[16] World Health Organization (WHO). “Solidarity” clinical trial for COVID-19 treatments. World Health Organization (WHO). Situation reports. Geneva: WHO. [Accessed: 5 Apr 2020]. Available from: https://www.who.int/emergencies/diseases/novel-coronavirus-2019/global-research-on-novel-coronavirus-2019-ncov/solidarity-clinical-trial-for-covid-19-treatments

[17] European Commission (EC). New research projects on Coronavirus (March 2020). Brussels: IC; 30 March 2020. Available from: https://ec.europa.eu/info/sites/info/files/research_and_innovation/research_by_area/documents/ec_rtd_cv-projects-1.pdf

[18] World Health Organization (WHO) Emergency Committee. Statement on the second meeting of the International Health Regulations (2005) Emergency Committee regarding the outbreak of novel coronavirus (2019-nCoV). Geneva: WHO; 30 January 2020. Available from: https://www.who.int/news-room/detail/30-01-2020-statement-on-the-second-meeting-of-the-international-health-regulations-(2005)-emergency-committee-regarding-the-outbreak-of-novel-coronavirus-(2019-ncov)

[19] World Health Organization (WHO) Emergency Committee. WHO Director-General's opening remarks at the media briefing on COVID-19 - 11 March 2020. Geneva: WHO; 11 March 2020. Available from: https://www.who.int/dg/speeches/detail/who-director-general-s-opening-remarks-at-the-media-briefing-on-covid-19---11-march-2020

[20] European Centre for Disease Prevention and Control (ECDC). Rapid risk assessment. Novel coronavirus disease 2019 (COVID-19) pandemic: increased transmission in the EU/EEA and the UK – sixth update. Stockholm: ECDC; 12 March 2020. Available from: https://www.ecdc.europa.eu/sites/default/files/documents/RRA-sixth-update-Outbreak-of-novel-coronavirus-disease-2019-COVID-19.pdf

[21] European monitoring of excess mortality for public health action (EuroMOMO). European mortality bulletin week 13, 2020. Copenhagen: EuroMOMO; April 2020. Available from: https://www.euromomo.eu/index.html

