# Supplementary figures and images for: A hundred days into the coronavirus disease (COVID-19) pandemic
Source: Euro Surveill. 2020 Apr 9;25(14):2000550. doi: 10.2807/1560-7917.ES.2020.25.14.2000550 (PMC7160442; doi:10.2807/1560-7917.ES.2020.25.14.2000550)

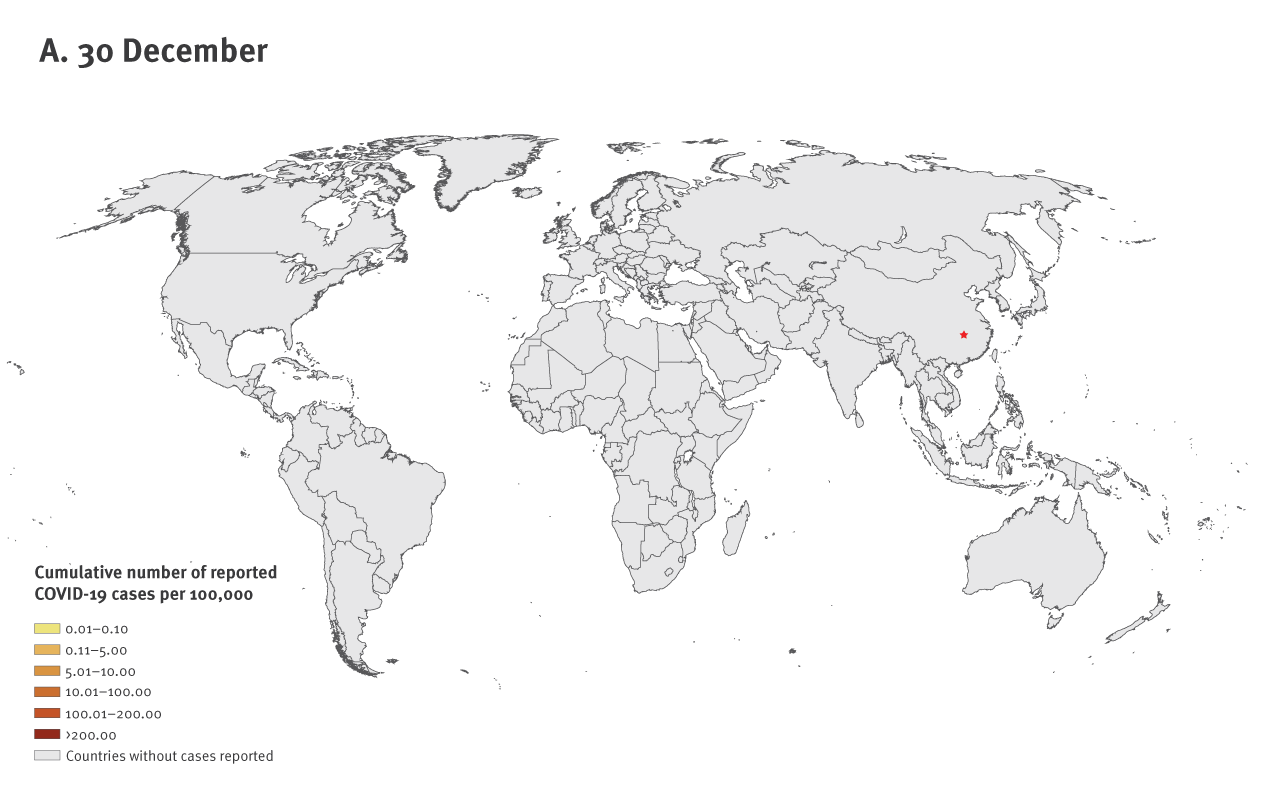

Supplement: Supplement [file 2000550_STEFFENS_Video_S1.gif]
